# Engineering a Pro-Osteogenic Secretome through the Transient Silencing of the Gene Encoding Secreted Frizzled Related Protein 1

**DOI:** 10.3390/ijms241512399

**Published:** 2023-08-03

**Authors:** Daniel García-Sánchez, Alberto González-González, Itzíar Álvarez-Iglesias, Mónica del Dujo-Gutiérrez, Alfonso Bolado-Carrancio, Matilde Certo, María Isabel Pérez-Núñez, José A. Riancho, José Carlos Rodríguez-Rey, Jesús Delgado-Calle, Flor María Pérez-Campo

**Affiliations:** 1 Department of Biochemistry and Molecular Biology, Faculty of Medicine, University of Cantabria-IDIVAL, 39012 Santander, Spain; daniel.garciasa@alumnos.unican.es (D.G.-S.); alberto.gonzalezgo@alumnos.unican.es (A.G.-G.); itziar.alvarez@alumnos.unican.es (I.Á.-I.); monica.deldujo@unican.es (M.d.D.-G.); matilde.certo@studenti.unimi.it (M.C.); josecarlos.rodriguez@unican.es (J.C.R.-R.); 2 Cancer Research UK Scotland Centre, Institute of Genetics and Cancer, University of Edinburgh, Edinburgh EH4 2XR, UK; boladoca@outlook.es; 3 Department of Traumatology, Hospital Universitario Marqués de Valdecilla, University of Cantabria, 39008 Santander, Spain; isabel.perez@unican.es; 4 Department of Internal Medicine, Hospital Universitario Marqués de Valdecilla-IDIVAL, University of Cantabria, CEBERER, 39012 Santander, Spain; jose.riancho@unican.es; 5 Department of Physiology and Cell Biology, Winthrop P. Rockefeller Cancer Institute, University of Arkansas for Medical Sciences, Little Rock, AR 72205, USA; jdelgadocalle@uams.edu

**Keywords:** mesenchymal stem cells, secretome, *Sfrp1*, osteogenesis, bone regeneration

## Abstract

The evidence sustaining the regenerative properties of mesenchymal stem cells’ (MSCs) secretome has prompted a paradigm change, where MSCs have shifted from being considered direct contributors to tissue regeneration toward being seen as cell factories for producing biotech medicines. We have previously designed a method to prime MSCs towards osteogenic differentiation by silencing the Wnt/β-Catenin inhibitor *Sfpr1*. This approach produces a significant increase in bone formation in osteoporotic mice. In this current work, we set to investigate the contribution of the secretome from the MSCs where *Sfrp1* has been silenced, to the positive effect seen on bone regeneration in vivo. The conditioned media (CM) of the murine MSCs line C3H10T1/2, where *Sfrp1* has been transiently silenced (CM-*Sfrp1*), was found to induce, in vitro, an increase in the osteogenic differentiation of this same cell line, as well as a decrease of the expression of the Wnt inhibitor *Dkk1* in murine osteocytes ex vivo. A reduction in the RANKL/OPG ratio was also detected ex vivo, suggesting a negative effect of CM-*Sfrp1* on osteoclastogenesis. Moreover, this CM significantly increases the mineralization of human primary MSCs isolated from osteoportotic patients in vitro. Proteomic analysis identified enrichment of proteins involved in osteogenesis within the soluble and vesicular fractions of this secretome. Altogether, we demonstrate the pro-osteogenic potential of the secretome of MSCs primmed in this fashion, suggesting that this is a valid approach to enhance the osteo-regenerative properties of MSCs’ secretome.

## 1. Introduction

The potential application of MSCs in regenerative medicine was initially focused on cell transplantation into the patients and the subsequent differentiation of those MSCs into a specific (resident) cell type to replace lost or damaged tissue. However, the different steps of cell manipulation required prior to the implantation of those cells into a given tissue has detrimental effects on cell viability. The need to expand in vitro the MSCs cells prior to implantation, to achieve a cell number sufficient to undergo many of the procedures, also increases genomic instability having a negative effect on the regeneration potential of those cells. Moreover, the administration route, such as local or systemic injection, can exert important mechanical stress on the cells, also limiting their viability.

In addition, current evidence in animal models shows that the beneficial effects of MSCs in regenerative therapies are achieved primarily through their important paracrine action on the bone marrow (BM) microenvironment rather than through their ability to differentiate into various cell lineages [1,2]. Bone marrow MSCs (BM-MSCs) secrete a range of bioactive products into the surrounding microenvironment, which have trophic effects on neighboring cells stimulating numerous biological processes [3,4]. The soluble factors and extracellular vesicles (EVs) produced by BM-MSCs are considered the main modulators of cellular crosstalk at the BM microenvironment, greatly influencing bone homeostasis and enhancing bone repair and regeneration [5,6]. These findings prompted a change in paradigm, with the researchers focusing their attention on the secretome of MSCs as a key tool for MSCs-mediated regenerative medicine.

Although the secretome from MSCs has broad proangiogenic, antifibrotic, anti-inflammatory, and antiapoptotic properties, its plasticity allows us to engineer the composition of the soluble fraction as well as that of the exosomal cargo to produce a tailor-made product that would fit specific therapeutic requirements [7]. Different approaches have been developed to engineer an MSCs-derived secretome or exosomal cargo for improving bone regeneration, from pre-conditioning MSCs with different biochemical compounds, small molecules or cytokines, to subjecting the MSCs to diverse biophysical cues or to directly altering MSCs gene expression through genetic manipulation. 

Recently, our team has been developing a method for the systemic management of osteoporosis based on the silencing of inhibitors of the main osteogenic signaling pathways in MSCs [8,9]. Silencing of target genes is achieved by using a particular type of Lock Nucleic Acid Antisense Oligonucleotides, known as GapmeRs which, in our system, are encapsulated in hybrid nanoparticles [8]. These nanoparticles are then delivered to the endogenous MSCs in the BM of a murine osteoporotic model, using an aptamer specifically designed to target these cells [10]. Although the use of the GapmeRs produces only a transient silencing of the target genes, this seems to be enough to prime MSCs towards osteogenic differentiation. In fact, using this method, which transiently silences *Sfrp1*, an antagonist of the Wnt/β-catenin signaling pathway, at the level of the endogenous bone marrow MSCs results in a significant increase in bone mineral density in an osteoporotic mouse model [10]. 

In the same way that MSCs potential to differentiate into osteoblasts can be increased by the transient silencing of anti-osteogenic genes, it is also possible that these modifications can alter their secretory profile to change the production of different factors, generating a secretome that has an overall positive effect on all the cells in the BM microenvironment. Hypothetically, the priming process, consisting of the transient silencing of key osteogenic inhibitors, would encompass important changes in gene expression and protein production. The secretome of the primed cells would likely include osteogenic proteins that are synthesized in the cytoplasm and later released outside the cell. Similarly, during their biogenesis, EVs such as exosomes, key constituents of the secretome, would capture and encapsulate cytoplasmic components, including specific proteins and RNA molecules linked to osteogenic differentiation, overall leading to the production of a secretome enriched in osteogenic factors. This secretome could have important paracrine effects on neighboring cells, including those responsible for bone homeostasis. That is, a cell primed for osteogenic differentiation would theoretically produce a more osteogenic secretome that could have similar osteo-regenerative properties than the modified cell. 

In this current work, we set out to investigate the effects of the secretome of MSCs where *Sfrp1* has been transiently silenced on other cells resident in the BM with a key role in bone homeostasis and further analyze the protein composition of the soluble and vesicular fractions of this secretome to identify the molecules with putative osteo-regenerative properties.

Although there are still limitations in MSCs-derived secretome-based therapies, various methods to engineer secretomes with increased osteo-regenerative potential could open a promising field in bone regeneration, which can attract further investigations.

## 2. Results

### 2.1. Assessment of the Pro-Osteogenic Effect of Conditioned Media from the Murine MSC Cell Line C3H10T1/2 Where Sfrp1 Expression Has Been Silenced (mCM-Sfrp1)

To test the pro-osteogenic effect of the conditioned media (CM) where *Sfrp1* has been transiently silenced using a specific GapmerR, we exposed the murine MSC line C3H10T1/2 to this CM for 48 h prior to start osteogenic differentiation. The CM produced by this approach, of murine origin, would be termed mCM-*Sfrp1* (hereafter). As a control, we used CM also produced in C3H10T1/2 cells previously transfected with a control GapmeR (mCM-Ctrl).

**Figure 1:**
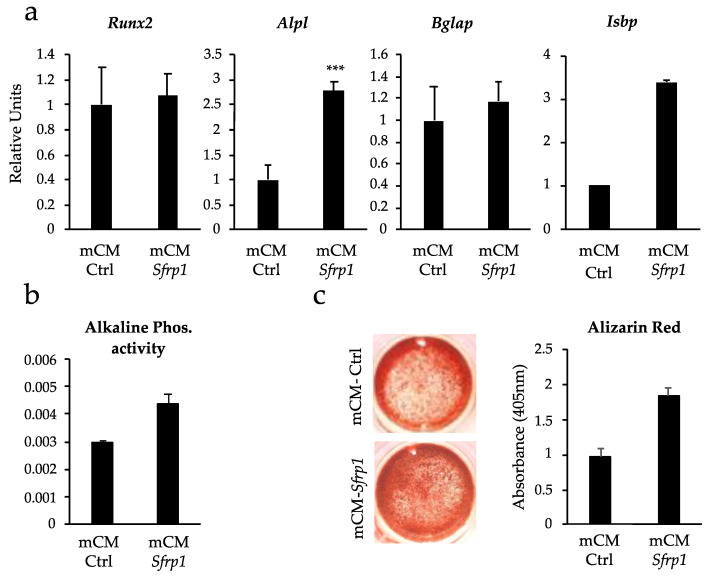
Effect of the CM from C3H10T1/2 cells where *Sfrp1* has been transiently silenced (mCM-*Sfrp1*) and control CM (mCM-Ctrl) on the osteogenic potential of the murine cell line C3H10T1/2. All analyses were performed at day 21 of osteogenic differentiation. (**a**) Semi-quantitative PCRs showing the relative expression levels of different osteogenic markers (*Runx2*, *Alpl*, *Bglap* and *Isbp*) pre-treated during 48 h with either the mCM-*Sfrp1* or mCM-Ctrl prior to the induction of osteogenic differentiation. Graphs represent average values of three independent experiments (*n* = 3). mCM-*Sfrp1* values are normalized to mCM-Ctrl values. (**b**) Graph showing the alkaline phosphatase activity in C3H10T1/2 under the different experimental conditions analyzed. Graph shows average values of three independent experiments (*n* = 3). (**c**) In vitro mineralization of C3H10T1/2 cells 21 days after being treated with the two CM revealed by Alizarin Red staining. mCM-*Sfrp1* values are normalized to mCM-Ctrl values. Results from one representative experiment out of three different experiments performed (*n* = 3). For all experiments, technical triplicates were also performed with each individual sample. In all graphs, bars represent standard error of the mean values (SEM). *** *p*-value < 0.0005.

Gene expression analysis of C3H10T1/2 cells treated with the mCM-Ctrl and mCM-*Sfrp1*, growing in osteogenic media for 21 days, showed important differences in the expression of key osteogenic markers (Figure 1a). Although at this particular timepoint we detected no changes in the expression levels of *Runx2* (Runt-related transcription factor 2), the main gene driving osteogenic differentiation, we did detect changes in three key osteogenic markers: Osteocalcin (*Bglap*), Alkaline Phosphatase (*Alpl*), and Integrin Binding Sialoprotein (*Ibsp*). In the case of *Bglap* and *Ibsp*, in all three experiments performed, there was always a trend towards an increase in the expression of this gene in cells treated with the mCM-*Sfrp1*, although these changes were never significant. However, expression levels of *Alpl* were significantly higher in cells treated with the mCM-*Sfrp1*. 

The increase in the *Alpl* expression levels correlated with the results obtained by quantifying the activity of this enzyme, which plays a critical role in mineralization (Figure 1b) where the CM-*Sfrp1* seems to stimulate this activity. Moreover, analysis of in vitro mineralization through Alizarin Red staining also showed an increase in mineralization in the cells pre-treated with the mCM-*Sfrp1*, underscoring the positive effect of this mCM-*Sfrp1* on promoting osteogenic differentiation in the C3H10T1/2 murine cell line. 

### 2.2. Effect of Conditioned Media from the Murine MSC Cell Line C3H10T1/2 Where Sfrp1 Expression Has Been Silenced (mCM-Sfrp1) on Murine Osteocytes

**Figure 2:**
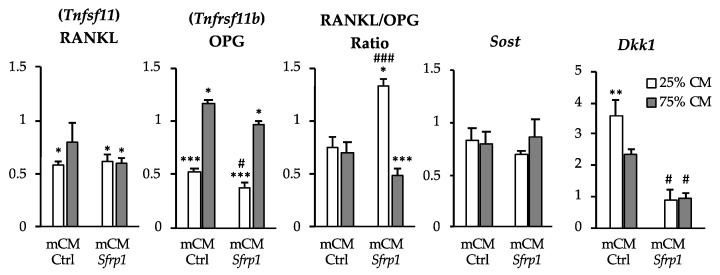
Effect of CM from the C3H10T1/2 cell line on the murine osteocytic cell line MLO-A5. Bars show gene expression in the osteocyte cell line MLO-A5 after being exposed for 48 h to different amounts (25% and 75%) of CM produced in the murine cell line C3H10T1/2 (mCM-*Sfrp1* or mCM-Ctrl). All values were normalized against the values obtained in cells growing in fresh media (value of 1). Values shown correspond to average values of three biological replicates (*n* = 3). In all graphs, bars represent standard error of the mean values (SEM). *t*-test vs. Fresh M *p*-value is represented with *. *t*-test mCM-*Sfrp1* vs. mCM-Ctrl *p*-value is represented with #. * and # *p*-value < 0.05; ** *p*-value < 0.005; ** and ### *p*-value < 0.005; *** and ### *p*-value < 0.0005.

As key cells in bone metabolism, we wanted to test the effect of the mCM-*Sfrp1* over murine osteocytes. This was completed by exposing the osteocytic murine cell line (MLO-A5) to different concentrations (25 and 75%) of mCM-*Sfrp1* and mCM-Ctrl for 48 h. As an additional control, we used fresh media. After this time, the expression of bone-related genes was quantified relative to the expression of those genes in cells grown in fresh media (value of 1). As key regulators of osteoclasts differentiation, we measured the expression of *Tnfsf11* and *Tnfrsf11b* encoding RANKL and OPG, respectively (Figure 2). The expression of *Sost* and *Dkk1,* both inhibitors of osteoblastic differentiation, was also measured (Figure 2). 

Except in the case of *Tnfrs11b*, the different concentrations of CM (25% and 75%) seemed to have similar effects on the MLO-A5 cell line. We detected an overall reduction in the levels of Tnfsf11, encoding RANKL, when cells were treated with either CM. Surprisingly, at the level of *Tnfrsf11b* expression, the 25% concentration of CM produced a significant decrease in the expression of this marker, compared to the fresh media, which was given a value of 1. To assess the global impact of the mCM-*Sfrp1* over osteocyte–osteoclast interaction, the RANKL/OPG ratio was also calculated (Figure 2). This ratio showed the overall availability of osteocyte-derived RANKL, with levels lower than 1, which corresponds to the value of cells grown in fresh media with no CM added. Our observations showed an overall reduction of RANKL availability with the mCM-*Sfrp1*, but only with the 75% concentration of CM. 

Although not significant, an overall tendency to the reduction in *Sost* expression was observed in all the experiments compared to the values obtained in cells growing in fresh media, with a value of 1. Importantly, while *Dkk1* expression seemed to be increased in cells treated with mCM-Ctrl, cells grown in the presence of mCM-*Sfrp1* did not show this increase, showing similar levels to that of the osteocytes grown without CM.

### 2.3. mCM-Sfrp1 Has a Positive Effect on Bone Regeneration Ex-Vivo

**Figure 3:**
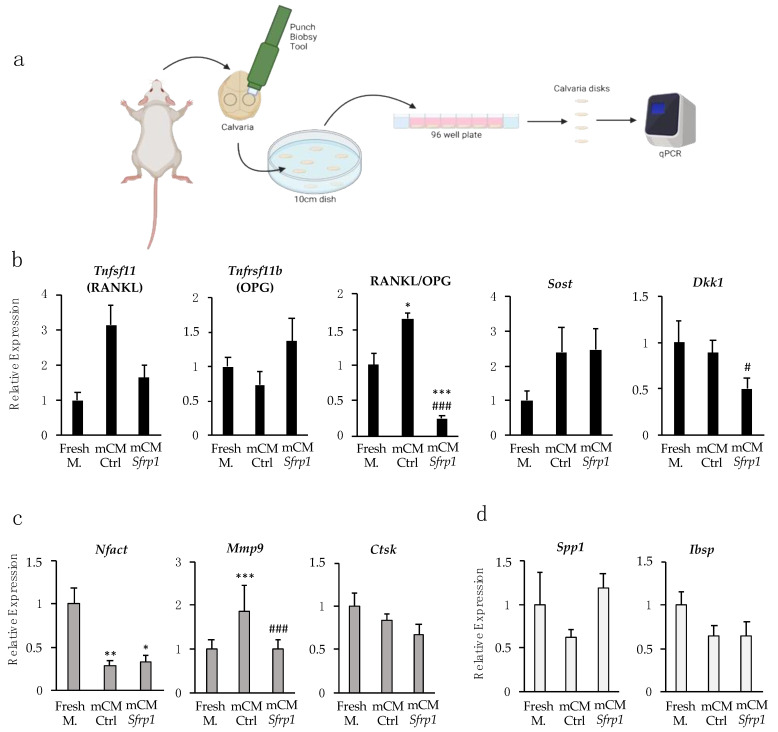
Gene expression of bone turnover markers in ex vivo cultures of murine calvaria bone treated with mCM-Ctrl y mCM-*Sfrp1.* (**a**) Experimental design. Gene expression analysis was performed from mRNA extracted from all calvarial disks after 11 days exposed to a 50% CM. mRNA was extracted and converted to cDNA prior to gene expression analysis by real time-PCR. The entire experiment was repeated three times. Each time, six calvarial bone discs were analyzed for each experimental condition (Fresh M, mCM-Ctrl and mCM-*Sfrp1*) (**b**) Expression of osteocytes related genes normalized to Fresh M. (**c**) Expression of osteoclast-related genes normalized to Fresh M. (**d**) Expression of osteoblast-related genes normalized to Fres M. In all graphs, error bars represent standard error of the mean values. *t*-test mCM-*Sfrp1* vs. Fresh M. *p*-value is represented with *. *t*-test mCM-*Sfrp1* vs. CM-Ctrl *p*-value is represented with #. * and # *p*-value < 0.05; ** *p*-value < 0.005; *** and ### *p*-value < 0.0005.

Ex vivo culture of mouse calvaria bone provides a system that preserves key cell-to-cell and cell-to-matrix interaction, allowing a more in-depth study of the effect of our secretome on bone cells in their natural microenvironment, where the complex interactions between these cells can be reproduced. Taking this into account, we set out to study the effect of CM-*Sfrp1* on all bone cells in an ex vivo culture of calvaria bone discs obtained from the mouse strain CD1. Bone discs were maintained in culture with 50% CM, either mCM-*Sfrp1* or mCM-Ctrl for 11 days. As control, calvarial discs were also maintained in media without CM added (fresh media). The results of fresh media were used for further normalization. After this time, bone tissue was disaggregated, and the total RNA was extracted in order to quantify the expression of different bone markers (Figure 3a). We tested osteocytic markers, including *Tnfsf11* (RANKL), *Tnfrsf11b* (OPG), *Sost*, and *Dkk1*; osteoblasts mineralization-related genes such as Osteopontin (*Spp1*) and Bone Sialoprotein (*Ibsp*); and finally, osteoclastic markers as the Nuclear Factor of Activated T cells 1 (*Nfatc1*), and bone remodeling and resorption-related genes as matrix metallopeptidase 9 (*Mmp9*), and cathepsin K (*Ctsk*).

Interestingly, while in the in vitro experiments with the osteocytic cell line, the CM from MSCs transfected with a non-specific GapmeR (mCM-Ctrl) showed a tendency to reduce the RANKL/OPG expression ratio, this CM seemed to have an opposite effect in the more complex microenvironment of the ex vivo culture, as the RANKL/OPG expression ratio was significantly upregulated. However, the RANKL/OPG ratio was significantly downregulated in calvarial bone disks treated with mCM-*Sfrp1* when compared with the control group (mCM-Ctrl) and the group treated only with fresh media. The expression of *Sost*, encoding esclerostin, shows a tendency to increase ex vivo in the presence of both CM, but in this case, results were not significant. Importantly, the expression of the Wnt pathway inhibitor *Dkk1* was significantly downregulated by the presence of the mCM-*Sfrp1* (Figure 3b).

On the other hand, the CM seems to have an overall inhibitory effect over osteoclasts, as suggested by a significant reduction in the expression of *Nfatc1* gene, encoding the osteoclastic transcriptional factor NFACT1, in both mCM-Ctrl and mCM-*Sfrp1* groups. There was also a similar tendency in the expression of *Ctsk*, related to bone remodeling and resorption. However, mCM-Ctrl showed a three-fold increase of the expression of the remodeling protein MMP9 coding gene, upregulation that was not observed in bone samples treated with mCM-*Sfrp1* (Figure 3c).

Regarding the osteoblastic genes, we observed no significant changes in the two osteogenic markers analyzed, *Spp1* and *Ibsp1* (Figure 3d).

### 2.4. hCM-SFRP1 Has a Positive Effect on the In Vitro Mineralization of Primary hMSCs from Osteoporotic Patients

Due to the positive effect of the mCM-*Sfrp1* on the osteogenic differentiation of the C3H10T1/2 murine cell line, we decided to test whether this CM had also a positive effect on the bone forming potential of MSCs from osteoporotic patients (hMSCs-OP), which are intrinsically characterized by a reduced osteogenic potential. Since we intended to test the effect of this CM on primary human MSCs, this time, we used a human mesenchymal stem cell line ASC57Telo to produce the CM instead of the C3H10T1/2 murine cell line. We needed to previously transfect this cell line with a GapmeR specifically designed to silence *SFRP1* expression in a human cell line. After testing that the expression of *SFRP1* has been correctly silenced (Supplementary Figure S1), we produced the CM in the human MSC cell line ASC57Telo using the same procedure as with the C3H10T1/2 murine cell line. hMSCs-OP were cultured in the presence of CM (hCM-*SFRP1* o hCM-Ctrl) for 48 h prior to the inducing differentiation with a specific osteogenic media.

**Figure 4:**
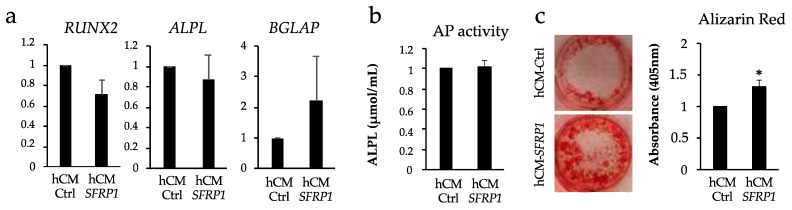
Effect of the CM from the human MSC cell line ASC57Telo where *SFRP1* has been transiently silenced (hCM-*SFRP1*) on the osteogenic potential of primary human osteoporotic MSCs (hMSCs-OP). All analyses were performed at 20 days of osteogenic differentiation. All graphs reflect average values from six different female patients with ages between 77 and 89 years old (*n* = 6). (**a**) Semi-quantitative PCRs showing the relative expression levels of different osteogenic markers: *RUNX2* (Runt Related Factor 2), Alkaline phosphatase (*ALPL*) and Osteocalcin, (*BGLAP*) pre-treated during 48 h with either the hCM-*SFRP1* or a CM from cells transfected with a control GapmeR (hCM-Ctrl). Technical triplicates were also performed with each individual sample. (**b**) Graph showing the average alkaline phosphatase activity in hMSCs-OP under the different experimental conditions analyzed. (**c**) In vitro mineralization of hMSCs-OP cells 20 days after being treated with the two conditioned media revealed by Alizarin Red staining. Results from one representative sample are shown in the pictures. Bar graph represents quantification of the staining. In all graphs, bars represent standard error of the mean values. * *p*-value < 0.05.

We used in total six hMSCs-OP extracted from the femoral head of osteoporotic patients. After a short period of in vitro expansion, hMSCs-OP were pre-treated with the hCM-*SFRP1* or hCM-Ctrl for 48 h before inducing osteogenic differentiation. The analysis at day 20 of differentiation did not show significant differences in the expression of the main osteogenic genes, although a trend towards a decrease in *RUNX2* expression levels and an increase in the expression levels of the osteogenic marker *BGLAP* were seen in all primary human MSCs (Figure 4a). No differences were detected either in the expression of *ALPL* or in the activity of this enzyme (Figure 4b). However, in agreement with an increased osteogenic capacity, Alizarin Red staining showed an important presence of calcium deposits in hMSCs-OP pre-treated with the hCM-*SFRP1* after 20 days of osteogenic differentiation compared to the same cells pre-treated with the hCM-Ctrl (Figure 4c).

### 2.5. Profiling MSCs Secretomes by Mass Spectrometry

**Figure 5:**
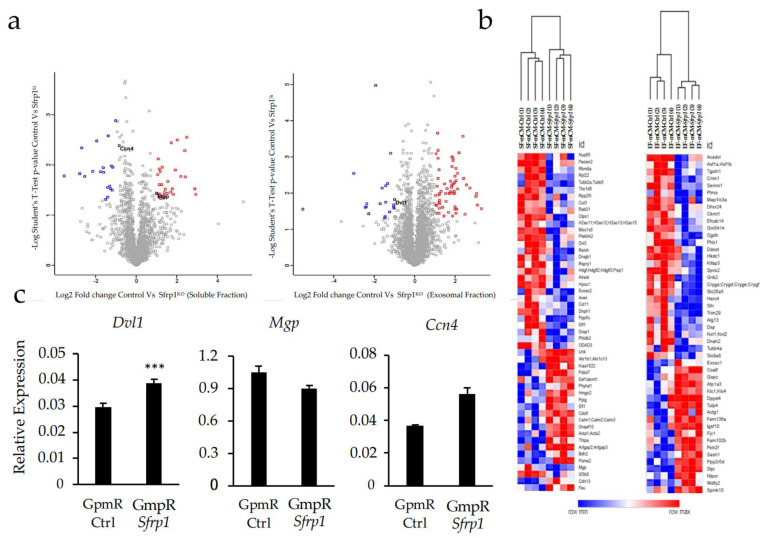
(**a**) Volcano plot showing changes in protein abundance in the different secretome fractions in mCM-*Sfrp1* compared with those of mCM-Ctrl. Both CM were obtained after transfecting the murine MSC line C3H10T1/2 with either a GapmeR for *Sfrp1* silencing or a Control GapmeR, respectively. Left: soluble fraction; right: exosomal fraction. (**b**) Image shows the hierarchical clustering of the significant hits found in the exosomal and soluble fraction. Left: soluble fraction; right: exosomal fraction. Hierarchical clustering was performed using Euclidean distance, and color shows the relative z-scored abundance of each hit among samples. In blue, statistically significant hits with two-fold enrichment in CM-*Sfrp1* compared to CM-Ctrl; in red, statistically significant hits with two-fold enrichment in wild-type cells. Samples corresponding to soluble fractions are named with the suffix SF-CM. Samples corresponding to exosomal fractions are named with the suffix EF-CM. (**c**) Validation of three of the hits by qPCR. *Dvl1* (Segment Polarity Protein Dishevelled Homolog DVL-1) is over-represented in the exosomal fraction of CM-*Sfrp1*, *Mgp* (Matrix Gla Protein) is under-represented in the soluble fraction of CM-*Sfrp1,* and Ccn4 (Cellular Communication Network Factor 4) is over-represented also in the soluble fraction of CM-*Sfrp1*. Graphs represent average values of three independent experiments (*n* = 3). mCM-*Sfrp1* values are normalized to mCM-Ctrl values. For all three experiments, technical triplicates were also performed with each individual sample. In all graphs, bars represent standard error of the mean values (SEM). *** *p*-value < 0.0005.

Mass spectrometry (MS) analysis of the changes induced in the secretome by the transient silencing of *Srfp1* in the murine mesenchymal stem cell line C3H10T1/2 revealed several proteins to be affected both in the soluble (Figure 5a left and 5b left) and exosomal (Figure 5a right and 5b right) fractions. Some of these proteins were known regulators of bone formation through different mechanisms. We selected three of those proteins with key roles in bone formation for validation by qPCR. The selected proteins were Dishevelled Segment Polarity Protein 1 (DVL1), which appeared to be more abundant in the exosomal fraction of the CM-*Sfrp1*, Matrix Gla Protein (MGP), with a higher presence in the CM-Ctrl in our MS analysis, and Cellular Communication Network Factor 4 (CCN4), which was over-represented in the soluble fraction of CM-*Sfrp1*. In all three cases analyzed, our results agreed with the relative abundance of those proteins found in the MS analysis. 

## 3. Discussion

Many works have shown that the number of transplanted MSCs that can contribute to tissue regeneration is minimal. Due to their low engraftment rates, it seems now clear that the therapeutic effect of MSCs is not only achieved by promoting the osteogenic differentiation of other MSCs [11,12] but most of the regenerative effects observed after MSCs implantation can be attributed to their paracrine action [5]. These results prompted a change in paradigm. In view of this, researchers have focused their attention on the secretome of MSCs as a key tool for MSCs-mediated regenerative medicine [13].

The bone microenvironment is complex, harboring many different cells that form an intricate communication network [14]. Even when focusing exclusively on the interactions related to bone homeostasis and remodeling, with the simplified osteocyte–osteoclast–osteoblast axis, external influences play a significant role, as they can modify the interactions within the axis, as seen in postmenopausal osteoporosis [15].

Here, we set out to investigate how the paracrine action of MSCs where *Sfrp1* has been silenced contributes to the increase in bone regeneration seen in an osteoporotic murine mouse model [10].

Regarding the analysis of the effect of the mCM-*Sfrp1* on osteoblasts, our experiments indicate a positive effect on osteoblast differentiation in vitro in the C3H19T1/4 murine cell line. However, the results using human primary osteoporotic MSCs were not as clear. We did observe a tendency to a reduction in the *RUNX2* expression levels in the presence of the hCM-*SFRP1*, which will be indicative of an accelerated osteogenic differentiation, as well as a trend to the increase of the late osteogenic marker *BGLAP*, although these data were not significant. This might be due to the small number of samples analyzed, which is directly related to the scarcity of samples that fit our stringent patient selection criteria in terms of sex, age, and medical history. The increase in the number of these samples would probably allow drawing more clear conclusions. However, the fact that we detected an increase in the levels of mineralized tissue formation in vitro when these cells are treated with the hCM-*SFRP1* would certainly suggest a positive effect on osteogenic differentiation. Although our in vitro results using the C3H10T1/2 cell line do point to a positive effect of the mCM-*Sfrp1* on C3H10T1/2 differentiation, we did not observe a significant effect of the mCM-*Sfrp1* over the analyzed osteogenic markers ex vivo. This might be explained by the relatively low presence of MSCs and osteoblasts in the calvaria bone discs relative to the presence of osteocytes, which are, by far, the most abundant cells in this kind of samples.

Osteocytes impact bone remodeling by secreting multiple factors that drive the differentiation and activity of other bone cells, mainly through the secretion of RANKL and OPG [16]. By expressing and secreting RANKL, osteocytes regulate the differentiation of the OCs precursors, which belong to the hematopoietic lineage [17]. OPG, however, acts as an antagonist to RANKL, inhibiting osteoclast differentiation by competing with RANKL for the binding to the RANK receptor. Therefore, the ratio between the expression of RANKL and OPG can be used as a marker of bone turnover. In our case, we did not detect a significant reduction of the RANKL/OPG ratio after incubating the osteocytic cell line MLO-A5 with the CM where *Sfrp1* was silenced (mCM-*Sfrp1*), but did observe a highly significant reduction of this ratio in the more complex microenvironment of an ex vivo culture of calvaria bone discs, when this has been treated with the mCM-*Sfrp1* compared to mCM-Ctrl. This suggests that this CM-*Sfrp1* might act on the osteocytes, modulating the production of RANKL and OPG in favor of the latter, therefore potentially contributing to decrease bone resorption previously observed in our osteoporotic murine models [10]. 

Osteocytes also act as regulators of their own precursors, the osteoblast. The most important factor in this regard is sclerostin, encoded by the *Sost* gene. Sclerostin, almost exclusively produced by osteocytes, is an important inhibitor of osteoblast activity and differentiation, as it blocks the Wnt/β-catenin signaling pathway by binding to the LRP4/5/6 membrane receptors. Similarly, DKK1, also secreted by osteocytes, competes with Wnt proteins for the binding to the LRP4/5/6 membrane receptors also inhibiting Wnt signaling [18]. Our in vitro analysis on the osteocytic cell line MLO-A5 showed that the treatment with the CM-*Sfrp1* does not lead to significant decrease in the expression of the Wnt pathway inhibitor sclerostin. However, the ex vivo culture showed an opposite effect, observing a trend to an increase of *Sost* expression, although this was again not significant. This variation between the in vitro and ex vivo experiments could be explained by the more complex microenvironment present in the ex vivo culture, where the interactions among different cells present in the bone sample interfere with the single effect of the CM on the osteocytes. Interestingly, *Dkk1* expression was also showed to be downregulated in the bone culture. This would indicate that the downregulation of *Dkk1* in osteocytes, in contact with the CM-*Sfrp1,* potentially contributes to the creation of a pro-osteogenic microenvironment. 

The effect of the CM on osteoclasts was also analyzed in the ex vivo culture. The analysis of the expression of osteoclastic markers showed a tendency towards an inhibitory effect. The expression of the main osteoclastic transcriptional factor *Nfatc1* was significantly reduced in both CM, and thus, this could not be linked to a specific effect of *Sfrp1* silencing on secretome composition. The expression of *Ctsk* also presented a tendency to decrease in both experimental groups. Interestingly, the expression of *Mmp9*, a proteolytic enzyme with a key role in bone resorption highly expressed in osteoclasts, was significantly downregulated in samples pre-treated with the mCM-*Sfpr1* suggesting a negative effect of the mCM-*Sfrp1* on the bone resorption process.

To further elucidate the mechanisms involved in the pro-regenerative effects of the secretome of C3H10T1/2 cells *Sfrp1* was silenced both the protein soluble fraction (SF) and protein present in the exosomal cargo were analyzed through mass spectrometry. Among the proteins significantly more abundant in the cargo of exosomes isolated from the mCM-*Sfrp1* (Exosomal fraction_EF), we found Segment Polarity Protein Dishvelled Homolog DVL-1 (DVL1). This result is not totally unexpected, as the silencing of *Sfrp1* and thus the sustained activation of the Wnt/β-catenin signaling pathway could certainly promote the expression of DVL1, since this protein is a key component of the Wnt/β-catenin signaling pathway that transduces the Wnt signal to downstream effector molecules [19]. Due to the mechanistic of the exosome biogenesis process, the higher presence of this protein in the cargo of the exosomes produced by cells where *Sfrp1* has been silenced would likely reflect a higher concentration of this protein in the cytoplasm of cells with a high activity of the Wnt/β-catenin signaling pathway and, thus, a higher osteogenic potential. Regarding the soluble fraction of this secretome, one of the proteins found to be significantly decreased in the soluble fraction of mCM-*Sfrp1* is the Matrix Gla protein (MGP). There is some discrepancy between the studies published up to this date regarding the role of this protein in bone regeneration. Some authors have shown that MGP has a role in promoting osteoblasts proliferation, differentiation, and mineralization [20]. However, our results would be at odds with these findings and in agreement with other works that point to MGP as a potent inhibitor of calcification. MGP would act on sequestering BMP, in particular BMP2, in vitro, therefore inhibiting osteogenic differentiation [21]. Interestingly, another protein also over-represented in the soluble fraction of the mCM-*Sfrp1* is CCN4. This protein characteristic of the bone extracellular matrix belongs to the CCN family, named from its founding members (Cyr61 CTGF, Nov, 6). CCN4 is typically found expressed in newly formed bone [22,23] and is found upregulated in healing bone after induced fracture, highlighting its key role in skeletal homeostasis [24]. Importantly, this protein is able to promote osteogenic differentiation in osteoprogenitor cells cultured in vitro when added exogenously [24,25]. Therefore, the significant enrichment of this protein in the secretome of MSCs where *Sfrp1* has been silencing could have a key role in promoting bone regeneration and might explain, at least in part, the effects seen in vitro in the C3H10T1/2 cells when exposed to the mCM-*Sfrp1*. 

Overall, the paracrine effect of endogenous MSCs on other cells in the bone marrow microenvironment, mainly on the osteocytes, after the inhibition of the Wnt antagonist *Sfrp1* in those cells, would overall enhance bone regeneration. This paracrine effect would add up to the already positive effect directly exerted by the silencing of *Sfrp1* in those MSCs, which would trigger their osteogenic differentiation. Therefore, the secretome of the modified cells would provide an added benefit to potential bone regeneration treatments. Our results suggest that the treated MSCs would be able to create a microenvironment that is not only pro-osteogenic, since it will promote MSCs differentiation to the osteoblastic lineage, but also anti-resorptive, by inhibiting osteoclast differentiation and reducing the available osteocyte-derived RANKL. 

The use of this pro-osteogenic secretome in cell-free therapies would allow us to bypass the need of directly silencing key regulatory genes at the endogenous MSCs by using LNA-ASOs, thus hypothetically reducing the potential side effects associated to the use of this molecules [26,27]. 

## 4. Materials and Methods

### 4.1. Cell Culture and Osteogenic Differentiation

Three different immortalized cell lines were used in this work. The murine MSC line C3H10T1/2 (Clone 8, Ref. CCL-226, ATCC, Manassas, VA, USA) and the human MSC line ASC52telo (Ref. SCRC4000, ATCC, Manassas, VA, USA) were cultured in Dulbecco’s Modified Eagle’s Medium, DMEM (Gibco Thermo Fisher Scientific, Waltham, MA, USA), supplemented with 10% FBS and 1% penicillin–streptomycin. In addition, ASC57telo needed supplementation with 0.2 mg/mL geneticin (G418 Sulfate, Corning, Manassas, VA, USA). Murine osteocyte cell line MLO-A5 [28] needed α-MEM culture media (Gibco Thermo Fisher Scientific, Waltham, MA, USA) supplemented with 2.5% FBS, 2.5% BCS (GE Healthcare Life Science, Logan, UT, USA), 1% penicillin–streptomycin,). For the MLO-A5 cells as these are non-adherent cells, all flasks and plates were coated with rat tail collagen.

For osteogenic differentiation of C3H10T1/2, 25.000 cells/cm^2^ were seeded and incubated overnight to allow attachment. Osteogenic induction was achieved 24h later by replacing the culture media with osteogenic media. Osteogenic media consist of DMEM supplemented with 20 mM β-glycerophosphate, 50 µM ascorbic acid, and 1 µM dexamethasone. For hMSCs-OP, osteogenic differentiation was performed in the same manner but using 20,000 cells/cm^2^.

### 4.2. GapmeR Delivery

For gene silencing, we used Locked Nucleic Acid Antisense Oligonucleotides (LNA-ASOs) from Exiqon (Qiagen, Venlo, The Netherlands). Specifically, we worked with GapmeRs, a particular type of LNA-ASOs with more resistance to degradation by endonucleases. For initial GapmeR delivery efficiency testing, we used Antisense LNA GapmeR Negative Control A (Ref. 339515 LG00000002-DDA) labeled with fluorescein (FAM) for flow cytometry detection. In addition, we also acquired specific GapmeRs for *Sfrp1* silencing in mouse (Ref. 339511 LG00219849-DDA) and human (Ref. 339511 LG00219859-DDA) cells.

The transfection protocol for C3H10T1/2 has been previously published [8]. Also, efficiency of *Sfrp1* silencing after C3H10T1/2 mouse cell line transfection with the indicated GapmeRs was verified in a previous work [9]. A similar transfection process was followed for the ASC57telo human cell line transfection. Briefly, 24 h after the C3H10T1/2 cell line was seeded at 12,500 cells/cm^2^ and ASC52telo at 15,000 cells/cm^2^, culture media was replaced by serum-deprived Opti-MEM I media (Invitrogen, Waltham, MA, USA). OptiMEM I media was mixed with the recommended amount of Dharmafect lipofection agent (Dharmacon, Horizon Discovery, Cambridge, UK). Separately, each GapmeR was also mixed with OptiMEM I media and incubated 5 min at room temperature (RT) prior to dropwise addition over Dharmafect mix. The final resulted mix was incubated for 20 min at RT and, subsequently, added dropwise to the seeded cells. Cells were incubated overnight at 37 °C. One volume of DMEM with 10% FBS and 1% penicillin–streptomycin was added to the wells and incubated at 37 °C for another 24 h.

### 4.3. Isolation of Human Mesenchymal Stem Cells 

Human MSCs (hMSCs) were isolated from femoral heads of female patients (ages from 77, 78, 80, 85, 87, and 89 years old) who suffer from osteoporotic fracture and needed hip replacement surgery. Cell isolation was carried out following previously described protocols [29]. These cells were cultured in MesenPRO RS Media (Thermo Fisher Scientific, Waltham, MA, USA) enriched with Glutamax and MesenPRO supplements and were left to form colonies and proliferate for 10–14 days at 37 °C and 5% CO_2_. Then, hMSCs were expanded to achieve a cell number of 7–10 × 10^5^. All patients gave informed consent, and the study protocol was approved by the Comité de Ética en Investigación Clínica de Cantabria.

### 4.4. Conditioned Media Production

To test the effect of the treated MSCs over other murine bone cells, conditioned media (CM) derived from MSCs previously transfected with specific GapmeRs was produced. For these, 12 million cells seeded in a 100 mm culture plate were transfected using non-specific GapmeR Ctrl and a GapmeR specific for *Sfrp1*. Forty-eight hours after transfection, the culture media was discarded, and after washing the cells once with PBS, it was replaced with a total of 12 mL of DMEM without FBS. Cells were then incubated for 48 h under normoxic conditions at 37 °C. 

Once the incubation was completed, the CM was collected and centrifuged at 400× *g* for 10′ at 4 °C, followed by another centrifugation of the supernatant at 1000 g for 10′ at 4 °C. Finally, the supernatant was collected and filtered through a 0.22 µm-syringe filter. The supernatant was aliquoted to avoid repeated freezing–thaw cycles and stored at −20 °C until needed.

To test the effect of CM-*Sfrp1* on MSCs from osteoporotic patients, the human cell line ASC57Telo was used. The CM production and collection is identical to that previously described for the murine cell line C3H10t1/2. The transfection method for these cells is described in Section 4.2.

### 4.5. Alizarin Red Quantification

Alizarin Red staining quantification was performed by adapting a previously described protocol [30]. Briefly, each well (24-well plate) was incubated for 30 min with 200 µL of 10% acetic acid with slight shaking. After the incubation, the plates were scraped and the whole volume together with the pellet was transferred into an Eppendorf tube. Tubes were then vortexed for 30 s followed by a 10-min incubation at 85 °C. Tubes were left to cool for 5 min on ice and then centrifuged at RT for 30 min at 13,000 rpm. Once the centrifugation was finished, a total of 150 µL of the supernatant was transferred into a new tube where 57 µL of 10N NaOH was added to each tube. Samples were then vortexed, and each tube was then plated into 2 wells of a 96-well-plate, 100 µL per well. Finally, the plate absorbance was measured at 405 nm. 

### 4.6. Alkaline Phosphatase Activity Quantification

In order to quantify the activity of the alkaline phosphatase enzyme activity, at the end point of the osteogenic differentiation, wells were washed once with PBS and incubated at RT for 5′ with 200 µL of Triton-X100 0.05% to allow cell lysis. Wells were then scraped, and each well content was transferred into an Eppendorf tube. Samples were subjected to 3 cycles of 30 s of sonication, while immersed in ice. Samples were then stored at −80 °C until further analysis. 

Enzyme activity quantification was performed by using P-nitrophenol as substrate following previously described methods [31].

### 4.7. Analysis of Osteogenic Genes Expression

mRNA was extracted from cell cultures and converted to cDNA as previously described (9) to perform gene expression analysis by real-time qPCR. These analyses were carried out using Taqman assays (Thermo Fisher Scientific, Waltham, MA, USA). For the mouse assays probes, references were as follows: *Gapdh* Mm99999915-g1, *Sfrp1* Mm00489161_m1, *Runx2* Mm00501578_m1, *Alpl* Mm01187117_m1, *Bglap* Mm03413726_m1. For the human assay probes, references were as follows: *GAPDH* Hs99999905_m1, *SFRP1* Hs00610060_m1, *RUNX2* Hs00231692_m1, *ALPL* Hs00758162_m1, *BGLAP* Hs01587814_g1.

### 4.8. Ex Vivo Bone Culture

To test the effect of the CM on the bone, ex vivo cultures of calvaria disks were isolated and cultured following a previously described protocol [32]. Briefly, calvaria bones were dissected from 12-week-old CD1 mice and cleaned of soft tissues. Two 5 mm bone disks were obtained from each calvaria using a biopsy punch (Ref. 22617, Kai Industries, Seki, Gifu, Japan). Bone disks were transferred into a 96-well plate, and 200 µL of media was added to each well. Bone discs were maintained in culture with 50% CM, both CM-Sfrp1 and CM-Ctrl for 11 days. After this time, bone tissue was disaggregated, and the mRNA was extracted in order to quantify the expression of different bone markers (Figure 3a). Different conditions of CM were tested, including: a control group without CM, a second group with 50% of mCM-Ctrl, and a third group with 50% mCM-*Sfrp1.*

Ex vivo culture was maintained for 11 days, and every 2–3 days half of the culture media was changed, maintaining the corresponding media of each condition. Bone disks were washed once with PBS and placed in 5 mL round bottom tubes containing 1 mL of TRIzol. The tissue was disaggregated with a T25 digital ULTRA-TURRAX (Ref. 0003725000, IKA-Werke, Staufen, Germany) dispersing instrument, with an S25N-8G (Ref. 0001024200, IKA-Werke, Staufen, Germany) dispersion tool. During tissue disaggregation, samples were kept on ice, and two disaggregation pulses of 20 s each were performed on each sample to avoid overheating. 

After disaggregation, the tubes were centrifuged at 2000 rpm for 5 min, and the TRIzol supernatant was collected. TRIzol was used for RNA extraction, followed by retrotranscription into cDNA and finally for gene expression quantification.

### 4.9. Secretome Analysis by Mass Spectrometry

To generate the conditioned media (CM), we used 2 × 10^6^ C3H10T1/2 cells that had been previously transfected with the Ctrl or the *Sfrp1* GapmeRs. Forty-eight hours after, the transfection media was replaced by DMEM without serum and CM production was allowed for 48 h. Conditioned medium was then collected and centrifuged 3 min at 1000 rpm at 4 °C, followed by second centrifugation at 3000 rpm for 10 min at 4 °C. Exosomal fraction was isolated using ultracentrifugation, 100,000 rpm 1 h at 4 °C in a Beckman ultracentrifuge (Optima L90-k). The supernatant was labelled as a soluble fraction and the pellet as exosomal fraction. The soluble fraction was concentrated using Amicon Ultra 3 KDa centrifugal filters. Both fractions were processed for mass spectrometry analysis using in solution digest protocol. In brief, proteins were denaturalized using 6 M Guanidine Hydrochloride, reduced and alkylate with 5 mM TCEP (tris(2-carboxyethyl)phosphine) and 10 mM Chloroacetamide, and sequentially digested with MS grade Lys-C and trypsin. Peptides were desalted and purified as previously described [33]. The eluted peptides were lyophilized in a Concentrator Plus (Eppendorf), resuspended in 0.1% TFA and analyzed by LC-MS/MS on a Orbitrap Fusion™ Lumos™ Tribrid™ Mass Spectrometer [34]. Protein identification and quantification was performed using the DIA-N/N software [35] and the Perseus software after values normalization by total protein in the cell culture. Statistical changes were determined using an adjusted *t*-test against control cells with a *p*-value 0.05 and a fold change of 2 or higher.

## Data Availability

The data presented in this study are available on request from the corresponding author.

## Supplementary Materials

The following supporting information can be downloaded at: https://www.mdpi.com/article/10.3390/ijms241512399/s1.
