# Supplementary figures and images for: Engineering a Pro-Osteogenic Secretome through the Transient Silencing of the Gene Encoding Secreted Frizzled Related Protein 1
Source: Int J Mol Sci. 2023 Aug 3;24(15):12399. doi: 10.3390/ijms241512399 (PMC10419110; doi:10.3390/ijms241512399)

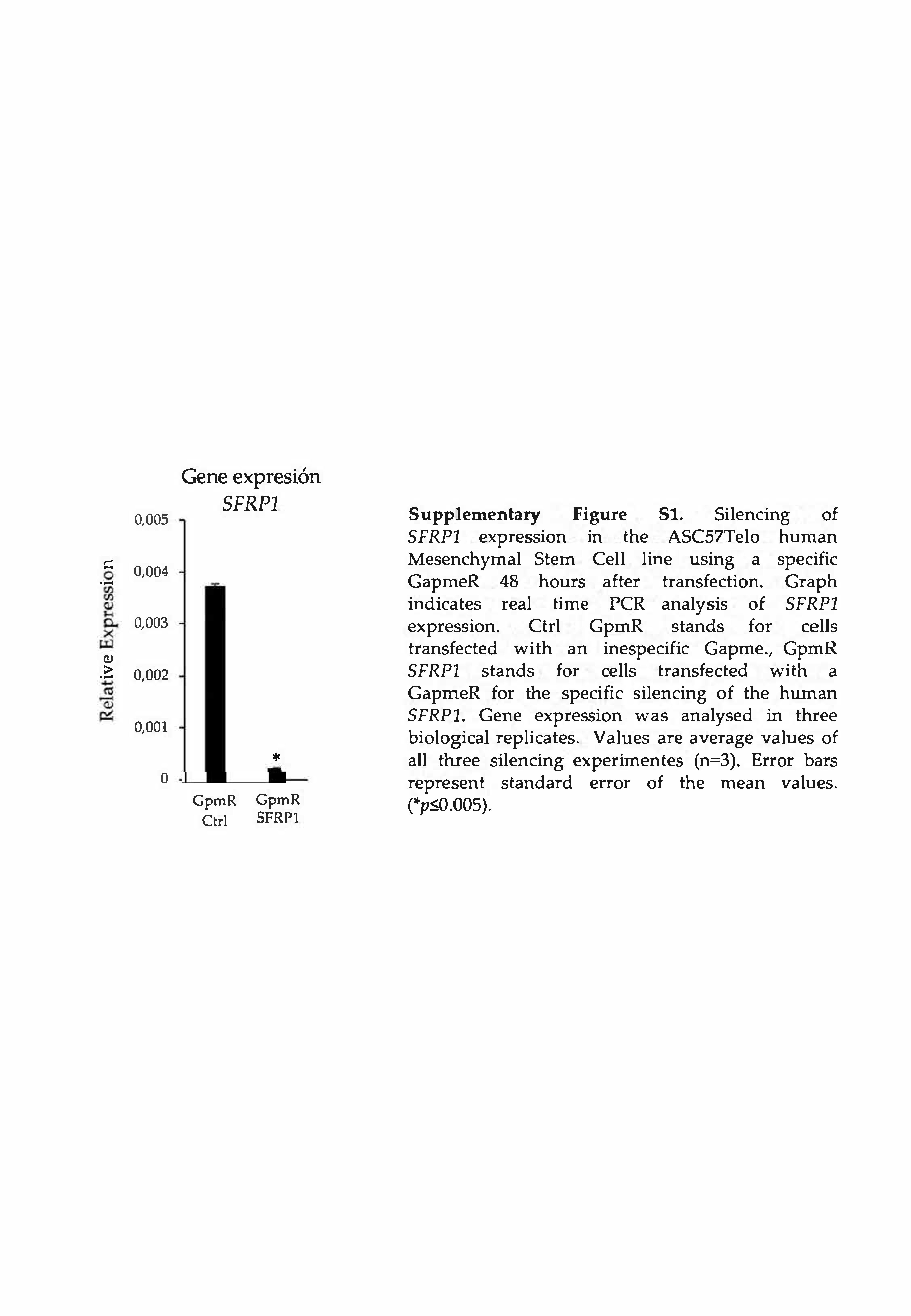

Supplement: Supplementary file 1 [file ijms-24-12399-s001.zip › ijms-2491870-supplementary.tiff]
